# DISYNCRO: Perceived roles of clinical study coordinators and data managers: results from a web-based survey of professionals from contract research organizations

**DOI:** 10.1016/j.conctc.2025.101533

**Published:** 2025-08-07

**Authors:** Susanna Yedro, Elena Tinari, Daniele Napolitano, Giulia Wlderk, Eleonora Ribaudi, Luciana Giannone, Gianluca Ianiro, Mattia Bozzetti, Antonio Gasbarrini, Vincenzina Mora

**Affiliations:** a CEMAD - Fondazione Policlinico Universitario A. Gemelli IRCCS, Rome, Italy; b Università Cattolica del Sacro Cuore, Rome, Italy; c ASST Cremona, Cremona, Italy

**Keywords:** “Clinical study coordinator”, **“**Data manager", "Clinical trials", "Contract research organizations"

## Abstract

**Introduction:**

The evolution of clinical trials has made it essential to introduce specific roles, such as Clinical Study Coordinator (CSC) and Data Manager (DM), into the research process. Their responsibilities sometimes overlap, creating operational challenges in the workplace. This study aims to determine how personnel at Contract Research Organizations (CROs) perceive the differences between the CSC and DM roles, assess their functional overlap, and identify areas where greater role clarity and training are needed to improve operational efficiency.

**Methods:**

An online survey instrument was used to gather data from CRO professionals through an internet-based questionnaire. The survey gathered sociodemographic data and included a knowledge assessment of 18 items and a 9-item role responsibilities section. Participants were stratified into three ability groups using Item Response Theory (IRT) analysis based on a Rasch model. McNemar's tests and non-parametric tests analyzed knowledge discrepancies and perceptual contradictions.

**Results:**

A total of 122 participants completed the survey. Most partecipants (98.4 %) identified the CSC as the primary figure within a research center, and 77.9 % considered the CSC essential for clinical trial execution. Regarding functional overlap, 57.4 % of respondents believed that the CSC could perform the duties of a DM, whereas only 42.6 % thought the DM could assume the CSC's responsibilities. Participants with lower levels of knowledge demonstrated a higher rate of contradictory responses, indicating greater difficulty distinguishing between the two roles.

**Conclusion:**

Study findings demonstrate an overwhelming preference for CSCs, who play a key versatile role in managing clinical trials. The insufficient theoretical understanding of the different duties of CSCs and DMs hampers operational efficiency. Establishing standard training programs combined with harmonization is essential to defining roles, enhancing teamwork, and providing quality clinical research practices.

## Highlights

• The survey investigated perceptions of CSC and DM roles in CROs.
• CSCs were viewed as crucial for clinical trial operations.
• Knowledge gaps regarding DM responsibilities were found.
• Higher ability levels correlated with greater role clarity and coherence.
• Standardized training is needed to improve understanding of the role in clinical research.

## 1 Introduction

Clinical trials have risen sharply over the past few years because of the demand for innovative treatments and solutions to current healthcare problems [1]. The rise in clinical research complexity has brought forth new professional positions, such as Clinical Study Coordinator (CSC) and Data Manager (DM), which are crucial for research support throughout the research process [2,3].

The execution of clinical trials involves multiple experts working together to contribute their distinctive expertise. It is important to note that role definitions for CSCs and DMs may vary significantly across settings, and there is no universally accepted distinction. This variability underscores the relevance of investigating how these roles are perceived in real-world Contract Research Organizations (CRO) environments. The CSC should guarantee compliance with international regulations, Good Clinical Practice (GCP) guidelines, and local and national regulatory requirements [4]. Nevertheless, in numerous clinical settings, the job description of CSC is poorly defined, poorly understood, and sometimes viewed as equivalent to that of DM. In some instances, one of the roles may be missing, which creates gaps in responsibility and supervision [2,5]. In the clinical research context, the lack of clear differentiation between CSC and DM roles can result in inefficiencies, including delays in query resolution and data cleaning [6,7]. These issues may compromise trial timelines and increase operational costs. Such challenges highlight the need for more explicit role definitions and targeted training to ensure that both professionals operate within their specific areas of expertise. Overlapping or duplicated duties may lead to confusion, inefficiency and reduced effectiveness. Identifying specific tasks for each role is crucial for optimizing the workflow, minimizing the number of errors, and guaranteeing the effectiveness and quality of clinical trial implementation [8]. Furthermore, the Declaration of Helsinki stresses the need for appropriate ethical and scientific training for research staff. It underlines the role of qualified supervision to guarantee the study's correctness and participants' safety [9].

The CSC position, introduced in the United States over two decades ago, has become a cornerstone of clinical trials management [10], although its responsibilities may vary across countries. In general, SCs are responsible for feasibility assessments, managing regulatory and contractual documentation, coordinating multidisciplinary teams, training personnel and serving as the primary connection between sponsors, CRO, Ethics Committees, and site staff [8]. Their participation is crucial in guaranteeing both the trial's success and efficiency. The DM role emerged more recently to address the increasing complexity of clinical data management. In this study, DM refers to professionals, typically CRO affiliated, who interact with clinical sites to manage trial data, rather than central DMs operating exclusively at the sponsor level. In the Italian context, it is uncommon for DM to be based directly at clinical sites. Instead, they typically operate from CROs or sponsor institutions and support site activities remotely, particularly in tasks such as data oversight and data validation [11]. Professionals in this position, typically with strong backgrounds in data science and informatics, are responsible for developing case report forms (CRF/eCRF), creating and maintaining the trial database, coding and cleaning the data, responding to queries, and ensuring that the information collected is accurate, consistent and complete [5,12]. The DM is crucial in the protection of data quality and the support of sound scientific analysis. Although CSCs and DMs work in different domains, they are both essential for clinical research success. Their cooperation guarantees scientific validity, effectiveness, and regulation compliance [13,14].

The CRO is another crucial actor in current clinical research, offering sponsors specialized operational and data management services. CROs assist in the planning and running trials, monitor GCP compliance, liaise with research centers, and oversee data collection and analysis [15]. As intermediaries between sponsors and clinical sites, CROs assist in strictly adhering to protocols and producing reliable and compliant data. Some of their tasks include documentation and preparation of regulatory documents, thus contributing to the overall credibility and success of the clinical research [16,17]. In this complex landscape, a clear and shared understanding of the roles and responsibilities of CSCs and DMs is essential to ensure effective collaboration and high-quality clinical trials execution. In the Italian context, the lack of formally established role definitions and national training standards for CSCs and DMs contributes to inconsistencies in operational practices and stakeholder expectations, particularly within CROs. Ambiguity and lack of standardization persist, complicating collaboration and role clarity. This study investigates how professionals working within CROs perceive the respective roles and responsibilities of CSC and DMs. In particular, it addresses the hypothesis that conceptual ambiguity, especially concerning the DM role, may hinder collaboration and operational efficiency in clinical trials. By identifying specific knowledge gaps and inconsistencies in role perception, the study aims to inform future training strategies and promote clearer professional boundaries between these two key roles.

## 2 Methods

### 2.1 Aim

The research investigates CRO personnel's views about the roles of the CSC and DM, evaluates their perceived task overlap, and identifies areas where insufficient role clarity may require targeted training and operational improvement.

### 2.2 Study design

This cross-sectional study, DISYNCRO, was designed to assess how CRO professionals perceive the CSC and DM roles. Between November and December 2024, the research team distributed a questionnaire through Microsoft Forms to CRO personnel.

### 2.3 Setting and data collection

The survey questionnaire reached CRO professionals who met two requirements: they were older than eighteen and worked as CRO professionals. The survey excluded participants who failed to consent to survey participation and data collection. Participants were recruited through targeted email invitations via CRO mailing lists, professional associations, and networks. No database sampling or participant pre-selection was applied. Built-in logic in the survey tool prevented missing data and duplicate entries. After data export, we manually reviewed responses to exclude incomplete questionnaires and verify consistency before analysis.

### 2.4 Data collection tool

The data collection instrument (Supplementary File) consisted of a structured questionnaire comprising 41 items, preceded by a brief introduction that outlines the study's objectives. The development of items was informed by literature review and expert opinion. Items 15–32 (Section 2) were based on role-specific competencies described in prior studies and guidelines. Meanwhile, Items 33–41 (Section 3) were designed to explore perceived operational distinctions between CSCs and DMs in everyday trial situations. The survey was organised into three main sections, each addressing different thematic areas.

### 2.5 Section 1 (items 1–14)

The survey gathered sociodemographic information (e.g., age, gender, education), occupational details about the participants' current and past work experience (e.g., their position in the CRO, years working in the sector, years in their current position, previous CSC experience), and their educational history (e.g., whether they received role-specific training, type and size of CRO).

### 2.6 Section 2 (items 15–32) quiz test

All participants received an 18-item knowledge test with binary scoring (correct/incorrect) that functioned as a quiz. Each participant received a total number of correct quiz answers, which became their QTOT score. The 18 knowledge questions used in this section are listed in Table 1. Table 1 presents abbreviated summaries of the quiz items; in the actual survey, these were administered in a dichotomous format (correct/incorrect). The proportion of correct responses for each item was computed to evaluate item-level performance. The Rasch model was employed to determine item difficulty by applying logit transformation of the proper proportion, which follows Item Response Theory (IRT) principles.

**Table 1:** Items difficulty - **Quiz Test**.

Question		Proportion (Correct)	Difficulty (Logit)
Q2	Who is the Data Manager?	0,582	−0,331
Q18	In Multidisciplinary Trials, who coordinates the various activities?	0,590	−0,365
Q15	Based on your experience, what is one activity that distinguishes the Clinical Study Coordinator from the Data Manager?	0,656	−0,644
Q6	Who is responsible for data entry at the site?	0,689	−0,793
Q9	Who ensures that the collected data is complete and accurate?	0,738	−1034
Q5	Who collaborates with the medical staff in managing the study proposal (protocol evaluation, feasibility, and related documents)?	0,762	−1165
Q7	Who is responsible for creating and managing the Case Report Form?	0,811	−1460
Q8	Who mainly communicates with the Ethics Committee for protocol approval requests?	0,885	−2043
Q16	What activities are commonly carried out by both the Data Manager and the Clinical Study Coordinator?	0,885	−2043
Q3	Who collaborates on-site with the Principal Investigator in managing protocol amendments?	0,967	−3384
Q17	How does the Clinical Study Coordinator handle a SERIOUS ADVERSE EVENT?	0,992	−4796
Q10	Who does the Clinical Study Coordinator primarily collaborate with?	0,992	−4796
Q11	What skills should a Data Manager have?	0,992	−4796
Q13	How important is it for the Clinical Study Coordinator to communicate with the research team?	1000	–
Q14	Who does the Clinical Study Coordinator regularly communicate with to ensure compliance with screening and randomization procedures?	1000	–
Q12	What skills should a Clinical Study Coordinator have?	1000	–
Q4	What is the main role of the Clinical Study Coordinator in conducting a trial?	1000	–
Q1	Who is the Clinical Study Coordinator?	1000	–

Note: Q, Question; Summary of knowledge quiz items used in Section 2 (presented in binary response format: correct/incorrect).

### 2.7 Section 3 (items 33–41) perceptions of the CSC or DM roles

This section contained dichotomous, closed-ended questions (e.g., yes/no or CSC/DM) to identify the particular role of the CSC in clinical trial operations. The topics include daily study coordination, clinical staff and patient communication and study protocol compliance monitoring. These questions are not scored, and responses will be analyzed in absolute terms and as percentages to accurately assess participants' views.

Although no formal pilot phase was conducted, the questionnaire was developed through iterative discussion among experts in clinical research methodology, and its internal structure was later assessed using Item Response Theory (IRT).

### 2.8 Data analysis

The analysis was structured by questionnaire section. Section 2 (knowledge items) was analyzed using IRT with a Rasch model to estimate item difficulty and participant ability. Section 3 (perception items) was analyzed using descriptive statistics and McNemar's tests to assess internal consistency and asymmetries in role perception. To obtain a more nuanced estimate of knowledge beyond raw scores, IRT was applied. IRT is a psychometric modelling approach that estimates item-level properties (such as difficulty) and respondent-level latent traits (ability). This study used a Rasch-model approximation (1 PL model), which assumes that the probability of a correct response is a logistic function of the difference between the person's ability and the item's difficulty.

Each participant's ability was quantified through a theta (θ) score, a continuous latent trait representing the individual's position on the knowledge continuum. Higher theta values indicate a greater probability of answering items correctly, thus reflecting higher ability levels. These theta scores were used to classify participants into three ordinal ability groups—Low, Medium, and High—based on quartile thresholds of the theta distribution.

Descriptive statistics were computed for all study variables. Continuous variables were summarised using central tendency and dispersion measures, while categorical variables were summarised using frequencies and percentages.

Group comparisons across ability levels were conducted using non-parametric inferential tests. The Kruskal-Wallis test was employed to assess differences in continuous variables. In contrast, Chi-square or Fisher's exact tests were used for categorical variables, depending on the size and distribution of cells. Where appropriate, pairwise Mann-Whitney U tests were used for post-hoc comparisons and effect sizes were estimated using rank-biserial correlation.

McNemar's tests were conducted on paired opinion items to examine participant responses' internal consistency and logical coherence. These analyses aimed to identify directional asymmetries and inconsistencies in participants' conceptualisations of role equivalence and functional redundancy. Additionally, a subset of participants who exhibited inconsistent response patterns was identified for exploratory subgroup analysis.

All analyses used Python (version 3.11) and standard scientific libraries. Statistical significance was set at *p* < 0.05.

### 2.9 Ethical considerations

The study used the principles of GCP and the Revised Declaration of Helsinki. Before enrollment, all participants received detailed oral and written information regarding the study's objectives and procedures and provided written informed consent. The study was approved by the Territorial Ethics Committee Lazio 3 (Approval N°0026951/24, dated November 06, 2024).

## 3 Results

A total of 144 individuals accessed the survey. Of these, 122 completed all required sections and met inclusion criteria; these participants were included in the final analysis. All 122 participants provided complete and valid responses in line with the inclusion criteria, and no exclusions were made after data cleaning. With 71.3 % of them being female, and the mean age was 36.0 years (SD = 7.2). Most respondents had a master's degree (59.8 %) and currently worked as Clinical Research Associate (CRA) (66.4 %). Most participants had worked in the CRO sector for 6.2 years on average and in their current position for 4.8 years, which suggests that the sample was relatively experienced and stable in their profession. Most respondents work in large CROs (>250 employees) and primarily work in pharmaceutical companies (90 %) that conduct phase III trials. The majority of them work in hybrid or remote work modes. Biotech companies and full-service CROs are less common. Phase IV trials are not very common, and neither are small companies. Phase II and on-site work are moderately standard (As shown in Table 2a, Table 2ba and 2b).

**Table 2a:** Socio-demographic and professional Characteristics by score quartile.

	Responders			
Variable	Low	Medium	High	Differences (p-value)
Age, M (SD)	37.3 (8.05)	35.32 (6.14)	36.09 (6.82)	0.560
Years in CRO sector	7.24 (6.21)	5.76 (5.04)	5.55 (5.36)	0.505
Years in current role	5.84 (5.64)	4.18 (4.54)	4.18 (2.18)	0.104
Gender (% Female)	70.9 %	71.4 %	72.7 %	0.976
Education level (≥Master's)	84.8 %	96.4 %	90.9 %	0.721
Previous experience as CSC	25.5 %	41.1 %	27.3 %	0.198
Role (CRA %)	85.5 %	83.9 %	81.8 %	0.721
Specific training received	90.9 %	80.4 %	63.6 %	0.198
Company type (CRO %)	69.1 %	62.5 %	63.6 %	0.375
Company size (large)	89.1 %	94.6 %	81.8 %	0.056
Therapeutic area (pharma)	90.9 %	82.1 %	100.0 %	0.173

Note: SD, Standard Deviation; CSC, Clinical Study Coordinator; DM, Data Manager; CRO, Contract Research Organization; CRA, Clinical Research Associate; Only the most relevant categories are reported. Percentages are calculated within each quartile.

**Table 2b:** Perceptions and attitudes toward CSC and DM roles by score quartile.

	Responders			
Variable	Low	Medium	High	Differences (p-value)
Total score, M (SD)	14.05 (1.34)	16.52 (0.50)	18.00 (0.00)	<0.001
Can the DM process data? (Yes)	69.1 %	82.1 %	90.9 %	0.186
Can the center work without CSC? (Yes)	0.0 %	1.8 %	9.1 %	0.290
Can the center work without DM? (Yes)	30.9 %	28.6 %	45.5 %	0.181
Who has better problem-solving skills? (CSC)	98.2 %	100.0 %	100.0 %	0.095
Can CSC do DM's work? (Yes)	74.5 %	64.3 %	45.5 %	0.544
Can CSCs do DM's work? (No)	74.5 %	82.1 %	81.8 %	0.541
Essential figure in a trial (CSC)	100.0 %	96.4 %	100.0 %	0.135
Preferred figure in a research center (CSC)	96.4 %	100.0 %	100.0 %	0.105

Note: SD, Standard Deviation; CSC, Clinical Study Coordinator; DM, Data Manager; Percentages refer to the proportion of respondents within each score quartile who gave the corresponding answer.

### 3.1 Section 2

The primary purpose of this study was to determine the views of the CRO staff on the roles of the CSC and DM and their differences in tasks and duties. The findings provide crucial information on the perceived clarity of roles and areas of convergence. All participants had a good understanding of the essential functions of the CSC in general. The results for the questions about the main tasks of the CSC (Q4, 100 % correct), teamwork with other team members (Q10, 99.2 % correct, logit = −4.796), and the management of serious adverse events (Q17, 99.2 % correct, logit = −4.796) were the highest. Items concerning the CSCs' interpersonal communication (Q13, Q14, 100 % correct) and the skills needed for the CSC (Q12, 100 % correct) also indicated that the role of the CSC is perceived as operational and relational in the clinical team. Conversely, lower accuracy was observed in items dealing with the differences in the tasks of CSC and DM. The question “Who is the DM?” (Q2) was found to have the lowest proportion of correct responses (58.2 %, logit = −0.331) on the DM's profile. Also, the items on coordination in multidisciplinary trials (Q18, 59.0 % correct, logit = −0.365), differences in specific tasks (Q15, 65.6 % correct, logit = −0.644), and who is responsible for data entry (Q6, 68.9 % correct, logit = −0.793) were also somewhat ambiguous. Intermediate values were obtained for items concerning feasibility support (Q5, 76.2 % correct, logit = −1.165), data verification (Q9, 73.8 % correct, logit = −1.034), and CRF management (Q7, 81.1 % correct, logit = −1.460). The question on the everyday tasks for CSC and DM also showed relatively high accuracy (88.5 % correct, logit = −2.043), though less than the more distinct CSC-related items (A shown in Table 1).

This pattern of item performance suggests that most questions are effective in distinguishing between different levels of knowledge. Still, some items may not have good discriminative power because of near-ceiling performance. This also strengthens the use of IRT analysis in assessing the test structure and improving the assessment tool. According to the IRT theta estimates, participants were categorized into three ability groups: low (n = 55), Medium (n = 56), and High (n = 11). These groups matched the lower (25), middle (50), and upper (75) quartiles of the theta distribution, as presented in Table 2a, Table 2ba and 2b.

### 3.2 Group comparisons

The analysis did not reveal any statistically significant differences across most demographic and professional variables, including gender, level of education, and current role. However, role-specific training was marginally significant (p = 0.0558), suggesting a possible relationship with higher ability. No differences were found in age or work experience. In contrast, both total quiz scores and ability estimates (thetas) were found to be significantly different across ability groups (p < 0.001), with all pairwise comparisons showing large effect sizes (r = ±1.0). The results showed that the participants in the high-ability group consistently outperformed those in the medium and low groups. (As shown in Table 2a, Table 2ba and 2b).

### 3.3 Section 3 - perceptions of the CSC and DM roles

Participants demonstrated a clear asymmetry in how they perceived the versatility and indispensability of the CSC and DM roles. While 91.0 % agreed that the two professionals have distinct tasks, a large majority (98.4 %) favored the presence of a CSC in the research center, and 77.9 % deemed the CSC indispensable for study execution. In comparison, only 61.5 % considered the DM equally essential. Additionally, 57.4 % believed that a CSC could fully perform the functions of a DM, whereas only 42.6 % thought the opposite. When asked to identify the most critical figure to ensure trial execution, 58.7 % selected the CSC. These findings underscore a perception of greater operational centrality and versatility associated with the CSC role.

To assess the internal coherence of perceptions regarding role distinctiveness, functional overlap, and professional indispensability, a series of McNemar's tests were conducted on key paired items. The results showed significant asymmetries across multiple comparisons. A significantly higher proportion of respondents considered the CSC indispensable compared to the DM (*p* < 0.001), and a greater number believed that the CSC could perform the tasks of the DM than vice versa (*p* < 0.001). Similarly, participants who perceived the roles as overlapping were more likely to state that the CSC could replace the DM than the reverse (*p* < 0.001), indicating an unbalanced view of role versatility.

**Fig. 1:**
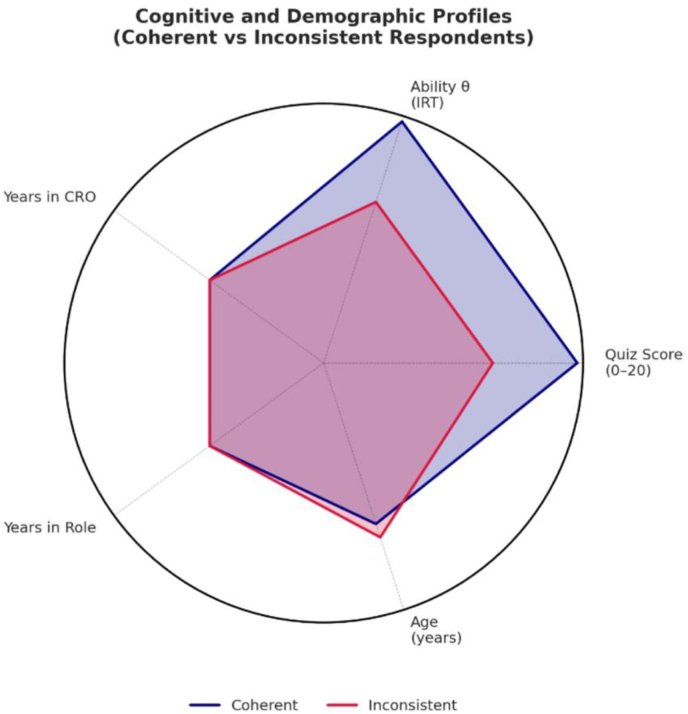
Radar chart of inconsistencies profiles.

Moreover, internal contradictions emerged between participants who viewed the roles as overlapping yet simultaneously rejected the idea that either role could fully replace the other. A small subgroup exhibited particularly incoherent response patterns; these individuals were characterized by significantly lower knowledge scores (*p* = 0.0015) (Fig. 1). Collectively, these inconsistencies point to conceptual confusion regarding task delegation and professional equivalence. Full results from the McNemar's tests are presented in Table 3.

**Table 3:** This table summarizes key pairwise comparisons examining perceptual inconsistencies in role understanding. Results from McNemar's tests are reported with p-values and brief interpretations to highlight conceptual asymmetries and training needs.

Comparison	p-value∗	Interpretation
CSC indispensable vs DM indispensable	<0.001	CSC seen as more essential
CSC can do DM tasks vs DM can do CSC tasks	<0.001	CSC perceived as more versatile
Role overlap vs CSC can replace DM	<0.001	Conceptual inconsistency in responses
Role overlap vs DM can replace CSC	0.001	Contradiction on DM replacing CSC
DM processes data vs DM indispensable	<0.001	DM role undervalued despite technical tasks
Overlap but neither can replace the other (inconsistents)	0.001	Lower ability linked to incoherence

Note: CSC, Clinical Study Coordinator; DM, Data Manager; ∗McNemar's Test.

## 4 Discussion

This study aimed to investigate how CRO professionals perceive the distinct roles of CSCs and DMs, assess the extent of functional overlap between them, and identify areas where insufficient role clarity may require targeted training to improve operational efficiency in clinical trials. In line with the study objectives, three key findings emerged as particularly significant. First, the CSC role was consistently perceived as more versatile and indispensable than the DM, with 98.4 % of respondents preferring a CSC in the research center and 77.9 % considering the CSC essential for clinical trial operations. Second, despite a formal recognition that CSCs and DMs have distinct responsibilities (91.0 %), a large proportion of respondents believed that CSCs could replace DMs (57.4 %). In comparison, fewer believed the reverse (42.6 %), highlighting a perceived asymmetry in functional capacity. Third, statistically significant inconsistencies in perceptions, captured through McNemar's tests, were more common among participants with lower knowledge levels. These internal contradictions suggest conceptual confusion regarding task delegation, role interchangeability, and the scope of data-related responsibilities. Collectively, these findings underscore the need for standardized training programs that clarify role boundaries and foster mutual understanding between complementary professional figures in clinical research.

This finding supports the study's hypothesis by showing that even though the DM's tasks are less explicit, the CSC is a well-known and crucial person in the clinical research context. While the strong recognition of the CSC's role may seem expected, it served as a functional benchmark within the study. This contrast enabled us to identify the relative conceptual gaps regarding the DM's responsibilities and to isolate the areas where clarification and training are most needed. The CSC position was perceived and aligned with high performance on all knowledge items. Most of the items that were used to assess the CSC's responsibilities, such as Q4 (100 %), Q10 (99.2 %, logit = −4.796), Q13 (100 %), and Q17 (99.2 %) had almost all participants answering correctly, thus reinforcing the CSC's flexibility, context applicability, and people management abilities in CROs.

On the other hand, questions on the DM's scope and responsibilities were not as well understood. For instance, only 58.2 % of the participants were able to define the role of the DM (Q2, logit = −0.331), and there was still confusion on the division of work (Q15, 65.6 %) and trial coordination in a multidisciplinary context (Q18, 59.0 %). These results reinforce the importance of the CSC in performing protocol-related activities and coordinating teamwork, cross-functional processes, and trial consistency – aspects perceived as more precise and appreciated by the respondents. The findings clearly show that the CSC is perceived as the mainstay of clinical trial operations and suggest a need to increase awareness, training, and the definition of the DM role to gain the same level of recognition and integration as the CSC. Furthermore, this study reveals that role ambiguity is still an issue in the Italian clinical research context, and that there is a lack of clarity on the different figures involved in the management of clinical trials, which may impede effective collaboration and performance, particularly when CRO professionals hold inconsistent expectations about role boundaries. This can lead to redundant task allocation, delayed data processing, or miscommunication with site staff, even when site-based CSCs clearly understand their role. Effective interdisciplinary collaboration requires clarity of the reciprocal role across all stakeholders involved [[18], [19], [20]]. Our findings confirm that functional boundaries between CSCs and DMs are not always clearly defined, reinforcing previous literature on the variability and evolving nature of these roles across organizational and national contexts. This is particularly relevant in Italy, where professional roles in clinical research are not yet fully institutionalized, and much of the task allocation is shaped by local practices rather than standardized competencies.

The results from knowledge scores showed that participants possessed good theoretical knowledge, yet Rasch modelling revealed distinct patterns between low-medium and high-ability groups. The high-ability group achieved either perfect or near-perfect scores. Still, the lower-ability group showed more significant response variability and produced inconsistent answers, particularly in the perception section. The conflicting responses, which included statements about CSC and DM roles being identical while denying their exchangeability, indicate a disorganized or unclear conceptual comprehension stemming from practical experience rather than formal education. The results showed that the “incoherent” subgroup performed significantly worse (p = 0.0015). The results confirm previous research [21], which indicates that unclear role comprehension in research teams can hinder effective task allocation and interprofessional communication, especially in complex clinical trials. Among less knowledgeable participants, such contradictions may also reflect difficulty in recognizing functional distinctions, leading to unrealistic expectations, role overlap, and reduced team clarity [10,22]. Although the notion that role ambiguity exists in clinical research is not new, our study contributes novel evidence by quantifying these gaps through Item Response Theory and highlighting how limited understanding—especially among lower-ability participants—translates into inconsistent perceptions of task delegation. Moreover, by focusing on CRO professionals rather than site-based staff, we provide insights into a stakeholder group that plays a key role in operational oversight but is less frequently investigated in this context.

The analysis revealed a near-significant relationship between role-specific training and ability level (p = 0.0558), which supports previous research about educational programs that enhance competence and role awareness. The academic program builds technical expertise while developing professional identity, role ownership, and teamwork capabilities. The analysis showed a statistically significant difference in the main clinical trial phase, including participants (p = 0.0291). The professionals working in Phase III trials showed higher operational complexity, leading to their placement in medium and high-ability groups. The results confirm previous research, showing that complex trial phases need better coordination, documentation, and communication abilities to build advanced professional competencies [14,23]. Conversely, those working in early-phase or post-marketing studies may have less exposure to such dynamics, limiting theoretical understanding and role differentiation [13,24]. Most participants (91.0 %) in the perception section agreed that CSCs and DMs have different functions. However, there was an asymmetrical view of their versatility: 57.4 % of the participants believed that the CSC could perform the duties of the DM, whereas only 42.6 % said the same for the DM. This perception aligns with the literature that describes the CSC as a "bridge role" that connects clinical, administrative, and regulatory tasks [8]. In contrast, the DM is seen as a highly specialized, technically focused figure whose essential contributions are often less visible in the day-to-day operations of a trial [25]. The CSC centrality is also supported by the fact that 98.4 % of the respondents preferred their presence in research centers, and 77.9 % considered them indispensable.

In comparison, only 61.5 % of the respondents considered the DM equally essential. Recent studies also support this view, pointing to the fact that the CSC is close to decision-making, has contact with patients, and is in touch with clinical staff, making them more relevant to the trial's success [26,27]. The role of DM in data integrity and scientific quality protection remains crucial even though some experts might underestimate this responsibility [28]. McNemar's test results also demonstrated directional differences in opinions about the interchangeability of roles. Most of the participants acknowledged the formal distinction between the two roles, but at the same time, believed the CSC could take over the DM responsibilities. Multiple studies [29,30] emphasize that unclear roles result in functional overlap, professional stress, inefficiency and communication breakdowns. This dynamic puts CSCs at risk of being overwhelmed while diminishing the technical value of DMs. The results of this research highlight the necessity of implementing specific training programs focused on clarifying the boundaries and interactions between these two roles. This lack of standardization has also been raised in national discussions on the role of clinical research professionals in Italy, although much of the existing literature remains descriptive or commentary-based [11]. National or European-level standardized training pathways could help address current knowledge gaps, enhance operational effectiveness, and improve the overall quality of clinical research.

The study contains certain methodological limitations. The research design and sampling strategy are monocentric and non-probabilistic, limiting the ability to generalize the findings to other settings. The online recruitment process and voluntary participation might have generated self-selection bias since it drew participants who were already familiar with or enthusiastic about the research subject. Most participants in the sample consisted of CRAs, which might have biased their perceptions and decreased the overall representation of different CRO roles. The low number of participants in the high-ability group (n = 11) reduces the statistical power for group comparisons.

Additionally, no formal sample size calculation was performed, as the study was exploratory. This limits the generalizability of findings and should be considered when interpreting the results. Moreover, the survey did not collect data on participants' prior experience as DMs, which may have limited our ability to analyse how direct DM experience influences role perception. The assessment relied solely on closed-ended questions in both the knowledge test and perception section, preventing the evaluation of participants' reasoning processes and complex viewpoints. The binary system of test response evaluation might not accurately measure the different levels of conceptual comprehension. The study has multiple research strengths despite certain methodological constraints. The research applied descriptive, non-parametric and psychometric (Rasch/IRT) methods to evaluate theoretical knowledge and perceptual coherence in detail. Rasch's modelling went beyond the raw score to accurately measure individual ability and item difficulty. Combining objective knowledge data with subjective perceptions delivered an extensive understanding of CRO personnel's understanding of the CSC and DM roles. The research method enabled researchers to identify conceptual ambiguities and explore the relationship between role perception and knowledge levels. The research provides new insights into national and international clinical research literature by concentrating on CRO personnel. The findings show the necessity to establish more explicit role definitions and to better recognise the complementary functions of CSCs and DMs in complex operational environments. Future research can draw on these findings to create training programs and supporting guidelines for enhanced clinical trial team structures.

The research showed stakeholders have a well-defined yet uneven understanding of CROs' CSC and DM functions. The CSC stands out as a fundamental and adaptable leader in trial administration, yet the DM faces limited recognition for its essential technical capabilities. The participants strongly preferred the CSC when evaluating potential role exchanges. The observed perceptions fail to demonstrate a thorough understanding of the underlying theory. The participants who showed lower knowledge about the subject matter gave inconsistent answers, which indicated their confusion regarding the unique duties of each position. Combining specific training and direct experience with later-phase clinical trials led to better conceptual understanding, highlighting the need for educational and practical training. Specifically, the survey results indicate that training should focus on clarifying the scope of data management responsibilities, the division of labor between CSCs and DMs, and the coordination of multidisciplinary teams. These were the areas where conceptual confusion and inconsistent perceptions were most evident, particularly among respondents in the low-ability group. The development of well-designed training programs stands as a crucial element in building a professional identity and enhancing collaboration between complementary roles. Establishing clear position responsibilities is essential for improving operational efficiency, avoiding role confusion, and protecting the quality of clinical research.

## CRediT authorship contribution statement

**Susanna Yedro:** Visualization, Investigation, Data curation. **Elena Tinari:** Writing – review & editing, Data curation, Conceptualization. **Daniele Napolitano:** Writing – review & editing, Writing – original draft, Project administration, Methodology, Formal analysis, Conceptualization. **Giulia Wlderk:** Writing – review & editing, Investigation, Data curation. **Eleonora Ribaudi:** Writing – review & editing, Project administration, Data curation. **Luciana Giannone:** Writing – review & editing, Methodology, Conceptualization. **Gianluca Ianiro:** Writing – review & editing, Validation, Supervision, Methodology, Conceptualization. **Mattia Bozzetti:** Writing – review & editing, Supervision, Formal analysis, Conceptualization. **Antonio Gasbarrini:** Writing – review & editing, Validation, Supervision, Resources. **Vincenzina Mora:** Writing – review & editing, Validation, Supervision, Project administration, Methodology, Conceptualization.

## Funding

This research did not receive any specific grant from funding agencies in the public, commercial, or not-for-profit sectors.

## Declaration of competing interest

The authors declare that they have no known competing financial interests or personal relationships that could have appeared to influence the work reported in this paper.

## Data Availability

Data will be made available on request.

## References

[1] Izarn F., Henry J., Besle S., Ray-Coquard I., Blay J.-Y., Allignet B. (2025). Globalization of clinical trials in oncology: a worldwide quantitative analysis. ESMO Open.

[2] Rico-Villademoros F., Hernando T., Sanz J.-L., López-Alonso A., Salamanca O., Camps C., Rosell R. (2004). The role of the clinical research coordinator--data manager--in oncology clinical trials. BMC Med. Res. Methodol..

[3] Riley D., Ward L., Young T. (1994). Oncology data management in the UK--BODMA’s view. British oncology Data Managers Association. Br. J. Cancer.

[4] Vijayananthan A., Nawawi O. (2008). The importance of Good Clinical Practice guidelines and its role in clinical trials. Biomed. Imaging Interv. J..

[5] Krishnankutty B., Bellary S., Kumar N.B.R., Moodahadu L.S. (2012). Data management in clinical research: an overview. Indian J. Pharmacol..

[6] Caminiti C., Maglietta G., Frau I., Peruzzotti G., Felisi M., van Dijk A. (2022). Presence and activities of clinical research coordinators at Italian Health Care Institutions: a national cross-sectional survey. J Clin Transl Sci.

[7] Mora V., Colantuono S., Fanali C., Leonetti A., Wlderk G., Pirro M.A., Palmarino F.M.C., Savini R., Ianiro G., Gasbarrini A. (2023). Clinical research coordinators: key components of an efficient clinical trial unit. Contemporary Clinical Trials Communications.

[8] Mora V., Colantuono S., Fanali C., Leonetti A., Wlderk G., Pirro M.A., Calà Palmarino F.M., Savini R., Ianiro G., Gasbarrini A. (2023). SC&RN group, clinical research coordinators: key components of an efficient clinical trial unit. Contemp Clin Trials Commun.

[9] General Assembly of the World Medical Association, World Medical Association Declaration of Helsinki: ethical principles for medical research involving human subjects, J Am Coll Dent 81 (2014) 14-18. 25951678

[10] Kengne Talla P., Robillard C., Ahmed S., Guindon A., Houtekier C., Thomas A. (2023). Clinical research coordinators' role in knowledge translation activities in rehabilitation: a mixed methods study. BMC Health Serv. Res..

[11] Cagnazzo C., Testoni S., Guarrera A.S., Stabile S., Taverniti C., Federici I., Pirondi S., Monti M. (2019). Clinical research coordinators: a crucial resource. Recent. Progress. Med..

[12] Nourani A., Ayatollahi H., Solaymani Dodaran M. (2022). Data management in diabetes clinical trials: a qualitative study. Trials.

[13] Getz K.A., Campo R.A. (2017). Trial watch: trends in clinical trial design complexity. Nat. Rev. Drug Discov..

[14] Peralta G., Sánchez-Santiago B. (2024). Navigating the challenges of clinical trial professionals in the healthcare sector. Front. Med..

[15] Roberts D.A., Kantarjian H.M., Steensma D.P. (2016). Contract research organizations in oncology clinical research: challenges and opportunities. Cancer.

[16] Beach J.E. (2001). Clinical trials integrity: a CRO perspective. Account. Res..

[17] Mirowski P., Van Horn R. (2005). The contract research organization and the commercialization of scientific research. Soc. Stud. Sci..

[18] Bozzetti M., Guberti M., Lo Cascio A., Privitera D., Genna C., Rodelli S., Turchini L., Amatucci V., Giordano L.N., Mora V., Napolitano D., Caruso R. (2025). Uncovering the professional landscape of clinical research nursing: a scoping review with data mining approach. Nursing Reports.

[19] Bozzetti M., Soncini S., Bassi M.C., Guberti M. (2024). Assessment of nursing workload and complexity associated with oncology clinical trials: a scoping review. Semin. Oncol. Nurs..

[20] Napolitano D., Lo Cascio A., Bozzetti M., Guberti M. (2025). Implementing research, improving practice: synergizing the clinical research nurse and the nurse researcher. Minerva Gastroenterol..

[21] Depp C.A., Howland A., Dumbauld J., Fontanesi J., Firestein D., Firestein G.S. (2018). Development of a game-based learning tool for applied team science communication in a virtual clinical trial. J Clin Transl Sci.

[22] Davis A.M., Hull S.C., Grady C., Wilfond B.S., Henderson G.E. (2002). The invisible hand in clinical research: the study coordinator's critical role in human subjects protection. J. Law Med. Ethics.

[23] Fujiwara N., Ochiai R., Shirai Y., Saito Y., Nagamura F., Iwase S., Kazuma K. (2017). Qualitative analysis of clinical research coordinators' role in phase I cancer clinical trials. Contemp Clin Trials Commun.

[24] Kozlowski S.W.J., Ilgen D.R. (2006). Enhancing the effectiveness of work groups and teams. Psychol Sci Public Interest.

[25] Bartolucci A., Jafar A.J., Sloan D., Whitworth J. (2019). Defining the roles of data manager and epidemiologist in emergency medical teams. Prehospital Disaster Med..

[26] Caminiti C., Maglietta G., Frau I., Peruzzotti G., Felisi M., van Dijk A. (2022). Presence and activities of clinical research coordinators at Italian Health Care Institutions: a national cross-sectional survey. J Clin Transl Sci.

[27] Fries R.A. (2002). Standard operating procedures for clinical research coordinators. Ther Innov Regul Sci.

[28] Zozus M.N., Lazarov A., Smith L.R., Breen T.E., Krikorian S.L., Zbyszewski P.S., Knoll S.K., Jendrasek D.A., Perrin D.C., Zambas D.N., Williams T.B., Pieper C.F. (2017). Analysis of professional competencies for the clinical research data management profession: implications for training and professional certification. J. Am. Med. Inf. Assoc..

[29] Larkin M.E., Lorenzi G.M., Bayless M., Cleary P.A., Barnie A., Golden E., Hitt S., Genuth S. (2012). Evolution of the study coordinator role: the 28-year experience in Diabetes Control and Complications Trial/Epidemiology of Diabetes Interventions and Complications (DCCT/EDIC). Clin. Trials.

[30] Speicher L.A., Fromell G., Avery S., Brassil D., Carlson L., Stevens E., Toms M. (2012). The critical need for academic health centers to assess the training, support, and career development requirements of clinical research coordinators: recommendations from the Clinical and Translational Science Award Research Coordinator Taskforce. Clin Transl Sci.

